# Swallowing kinematics and submental muscles activation during a newly designed maneuver called Mouth Open Swallowing Maneuver: A comparative study

**DOI:** 10.1371/journal.pone.0299845

**Published:** 2024-03-25

**Authors:** Ömer Faruk Yaşaroğlu, Selen Serel Arslan, Emre Cengiz, Rabia Alıcı, Numan Demir, Berna Oğuz, Tülin Düger

**Affiliations:** 1 Faculty of Physical Therapy and Rehabilitation, Hacettepe University, Ankara, Türkiye; 2 Department of Radiology, Hacettepe University Hospitals, Ankara, Türkiye; University Putra Malaysia, MALAYSIA

## Abstract

The aim of this study was to design a new maneuver called the Mouth Open Swallowing Maneuver (MOSM), and to compare swallowing kinematics and submental muscles activation (SMA) between MOSM and two current approaches used in dysphagia rehabilitation. Fifty healthy volunteers were asked to perform three repetitions of dry swallowing (DS) (control task), the MOSM, the Mendelsohn Maneuver (MM), and the Tongue-Hold Maneuver (THM) during videofluoroscopic swallowing study accompanied with simultaneous SMA recording. Swallowing kinematics were measured by frame-by-frame analysis on hyolaryngeal movement using ImageJ. Swallowing with maximum hyolaryngeal movement and SMA during these tasks was used for comparative analysis. Vertical movement of the hyoid during the MOSM was significantly greater than those observed during the DS and the THM (p<0.001, p<0.001). Horizontal movement of the hyoid during DS and the THM was significantly greater than that observed during the MM (p = 0.001, p = 0.001). Vertical movement of the larynx during the MOSM was significantly greater than those observed during DS, MM, and THM (p<0.001). There was no significant difference between tasks in horizontal movement of the larynx (p = 0.785). SMA during the THM was significantly greater than that observed during MOSM (p = 0.002). No significant difference was found between other tasks in terms of SMA (p>0.05). The MOSM as a newly designed maneuver was significantly superior to other maneuvers in increasing vertical hyolaryngeal movement. The THM has as much effect on hyolaryngeal movement as the MM. In this study, the MOSM was shown to be effective in increasing hyolaryngeal movement.

ClinicalTrials.gov Protocol Registration and Results System (PRS); the clinical trial registration number (NCT05579041).

## Introduction

Swallowing is a series of sequential functions performed by more than 30 muscles and nerves under control of cortical and subcortical centers. Oropharyngeal swallowing function mainly includes tongue-palate contact, tongue retraction, velopharyngeal valve elevation, protraction of the posterior pharyngeal wall, tilt of the epiglottis, adduction of the vocal folds, hyolaryngeal movement, and opening of the upper esophageal sphincter (UES) [1, 2]. Problems in one or more of these subfunctions cause swallowing disorders. Exercises and maneuvers in dysphagia rehabilitation are focused on improving these parameters [3]. Rehabilitation goals are determined according to the limitations and main problems of the patients with swallowing disorders [4]. Decreased muscle strength and endurance may cause inadequacy in physiological parameters such as tongue retraction, protraction of the posterior pharyngeal wall, and hyolaryngeal movement during swallowing [5]. Furthermore, timing problems can threaten swallowing safety with delayed initiation of swallowing and premature bolus spillage [4]. Therefore, patient-specific swallowing rehabilitation consists of training muscle strength and endurance, accelerating the initiation of swallowing, and adjusting the timing of the best bolus flow with controlled swallow or a combination thereof [4, 5].

One of the most critical events during pharyngeal swallowing is the hyolaryngeal movement that contributes to the closure of the airway and opening of the UES. Hyolaryngeal movement is the sum of anterior and superior movements of the hyoid and larynx. It is necessary to understand the vectorial forces generated by the muscles involved in the movement to better understand the hyolaryngeal movement. The submental muscles consisting of mylohyoid, geniohyoid, and anterior digastric muscles are positioned anteriorly between the hyoid and mandible. The posterior digastric, stylohyoid, and longitudinal pharyngeal muscles are located posteriorly. Anteriorly positioned muscle groups create anterior-superior and posteriorly positioned muscle groups create posterior-superior vectoral force for hyolaryngeal movement. Therefore, hyolaryngeal movement is achieved anteriorly and superiorly with the cooperation of the two muscle groups [6, 7]. A decrease in the amount and duration of hyolaryngeal movement affects the safety of swallowing by causing aspiration of food into the lung as it passes through the pharynx [8]. In addition, pharyngeal muscle weakness and impaired UES opening cause pharyngeal residue and impair swallowing efficiency [9].

Rehabilitation methods aim to increase the safety and efficiency of swallowing by focusing on strength, velocity, endurance, or their combination [10]. Exercise is an approach that causes long-term and permanent effects on swallowing physiology. Maneuvers have immediate or compensatory effects on swallowing physiology. Therapeutic effects, such as exercise, occur when maneuvers are learned and practiced for a long time [4]. There are some rehabilitation approaches known to improve hyolaryngeal movement such as the Mendelsohn Maneuver (MM), the Shaker exercise, and effortful swallow [11–13]. These are the most used approaches in dysphagia rehabilitation. However, there are few kinematic studies to explain their contribution to hyolaryngeal movement. The MM is the method of voluntarily holding the larynx elevated for a while during swallowing. It includes both strength and endurance components, and increases hyolaryngeal movement and UES opening [14]. Inamoto et al. found that the MM increased vertical movement of hyoid compared to normal swallowing [15]. Shaker exercise has a protocol that involves isometric and isotonic contractions with head lift to improve the width of the UES opening by increasing hyolaryngeal movement [16]. Effortful swallow was developed to enhance contact with the tongue base and posterior pharyngeal wall [17]. Additionally, Jang et al. reported that effortful swallow has a positive effect on increasing vertical hyolaryngeal movement [13]. The Tongue-Hold Maneuver (THM), also known as the Masako Maneuver, is performed by holding the tongue between the front teeth during swallowing. It provides a contact between tongue base and posterior pharyngeal wall by increasing anterior movement of the pharyngeal constrictors [18]. Functions of the pharyngeal muscles during the THM have been investigated, but its contribution to hyolaryngeal movement has not been investigated [19, 20]. The positioning of the tongue between the teeth creates a challenge for swallowing. Studies have reported that THM increases the risk of aspiration and pharyngeal residue and causes shorter airway closure and delay in the initiation of pharyngeal swallowing [18, 21]. Therefore, we believe that the hyolaryngeal kinematics would also be affected during this maneuver.

Rehabilitation approaches for hyolaryngeal movement in the literature are subject to certain conditions. The Shaker exercise cannot be used in surgeries that restrict cervical region movements or with the presence of the tracheostomy tube [22, 23]. In addition, several studies have shown that the tendency of muscles to fatigue can reduce the efficacy of the Shaker exercise [24, 25]. The MM and the THM (even if it contributes to the hyolaryngeal movement) require good cooperation. It may even be necessary to use biofeedback [26]. During therapy, patients have some difficulty in performing maneuvers that they are not familiar with or cannot directly visualize. In addition, voluntary control of the oropharyngeal muscles is expected in the patient during the maneuvers. Biofeedback increases control over the physiological process of swallowing by providing visual or auditory feedback to the patient [27]. Effortful swallow is considered as the easiest in terms of adaptation. There are few options when the patient is unable to perform any exercise or maneuver. Therefore, new alternative approaches are required so that the clinician could choose the appropriate exercise for each patient. The Mouth Open Swallowing Maneuver (MOSM) is a newly designed maneuver that focuses on increasing hyolaryngeal movement. The MOSM involves dry swallowing (without bolus) while a 10 mm wedge is comfortably placed on the molar teeth to keep the mouth open. In the hyolaryngeal complex, the mandible, hyoid, and larynx are closely related to each other through muscles and tendons. During mouth opening, the hyoid moves downward and backward with the depression of the mandible [28]. Therefore, a greater range of motion may be provided for hyolaryngeal movement. As the hyolaryngeal structure will move further, the load on the muscles may also increase. Muscle activation of the hyoid muscles, which are primarily responsible for hyolaryngeal movement, may increase as a result of this load. The effects of the MOSM on both swallowing kinematics (SK) and submental muscles activation (SMA) should be investigated. The aim of this study was to investigate SK and SMA of the MOSM by comparing with the MM and the THM used in dysphagia rehabilitation and dry swallowing (control task). The first hypothesis of the current study is that the MOSM maneuver elicits more hyolaryngeal movement than other tasks, and the second hypothesis is that the MOSM causes more SMA.

## Materials and methods

### Study design

**Fig 1:**
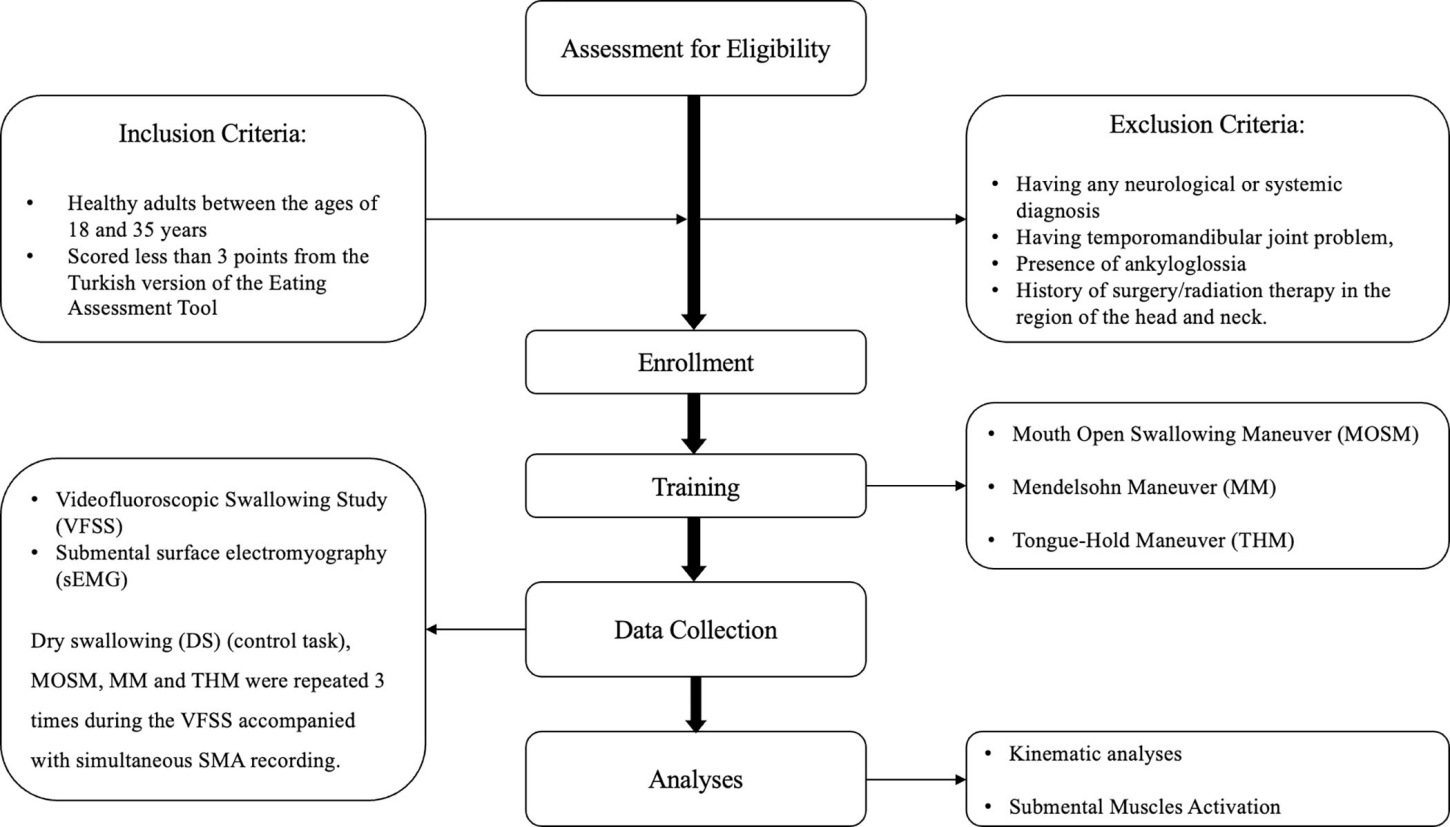
The flow chart of the methodology.

This prospective study was conducted at Hacettepe University Faculty of Physical Therapy and Rehabilitation and Department of Radiology at Hacettepe University Hospitals between 20 June 2021 and 15 November 2022. Ethical approval was obtained from Hacettepe University Clinical Research Ethics Committee (Decision number: KA-21039). Written informed consent forms were obtained from the participants. With the exception of the authors involved in data collection, other authors do not have access to information that could identify individual participants during or after data collection. The flow chart of the methodology is given in Fig 1.

### Participants

Healthy adults between the ages of 18 and 35 years who volunteered to participate in the study and scored less than 3 points from the Turkish version of the Eating Assessment Tool (T-EAT-10) were included in the study [29]. The T-EAT-10, which is an easy-to-use and practical instrument, is used to assess the dysphagia symptom severity. It has 10 questions, and each question is scored from ’0’ (no problem) to ’4’ (serious problem). Higher scores mean severe swallowing disorder. A score of 3 and above suggests the presence of swallowing disorder and a score of 15 and above suggests suspicion of aspiration [30]. Exclusion criteria were having any neurological or systemic diagnosis, temporomandibular joint problem, presence of ankyloglossia or a history of surgery/radiation therapy in the region of the head and neck.

### Swallowing tasks

Before data collection, participants were trained until they performed all the tasks correctly. Finally, we verified in the Videofluoroscopic Swallowing Study (VFSS) whether they performed the tasks properly. Participants started with dry swallowing first, and the other tasks were performed randomly during data collection. All tasks were repeated 3 times during the VFSS accompanied with simultaneous SMA recording. Therefore, a total of 12 swallows were recorded for each participant. Between tasks, the participants were allowed to drink as much water as they wanted to avoid dry mouth.

Participants performed 4 different tasks including dry swallowing, Mouth Open Swallowing Maneuver, Mendelsohn Maneuver and Tongue-hold Maneuver.

#### Dry swallowing (DS) (control task)

The maneuvers used in the study involve interventions that affect normal swallowing physiology. Therefore, dry swallowing was chosen as a control task to compare the effects of the maneuvers on kinematic and electrophysiological parameters of normal swallowing. Participants were asked to swallow their saliva in a neutral head and neck posture while in a sitting position. After the recording started, the participants were instructed to ‘swallow’.

#### Mouth Open Swallowing Maneuver (MOSM)

**Fig 2:**
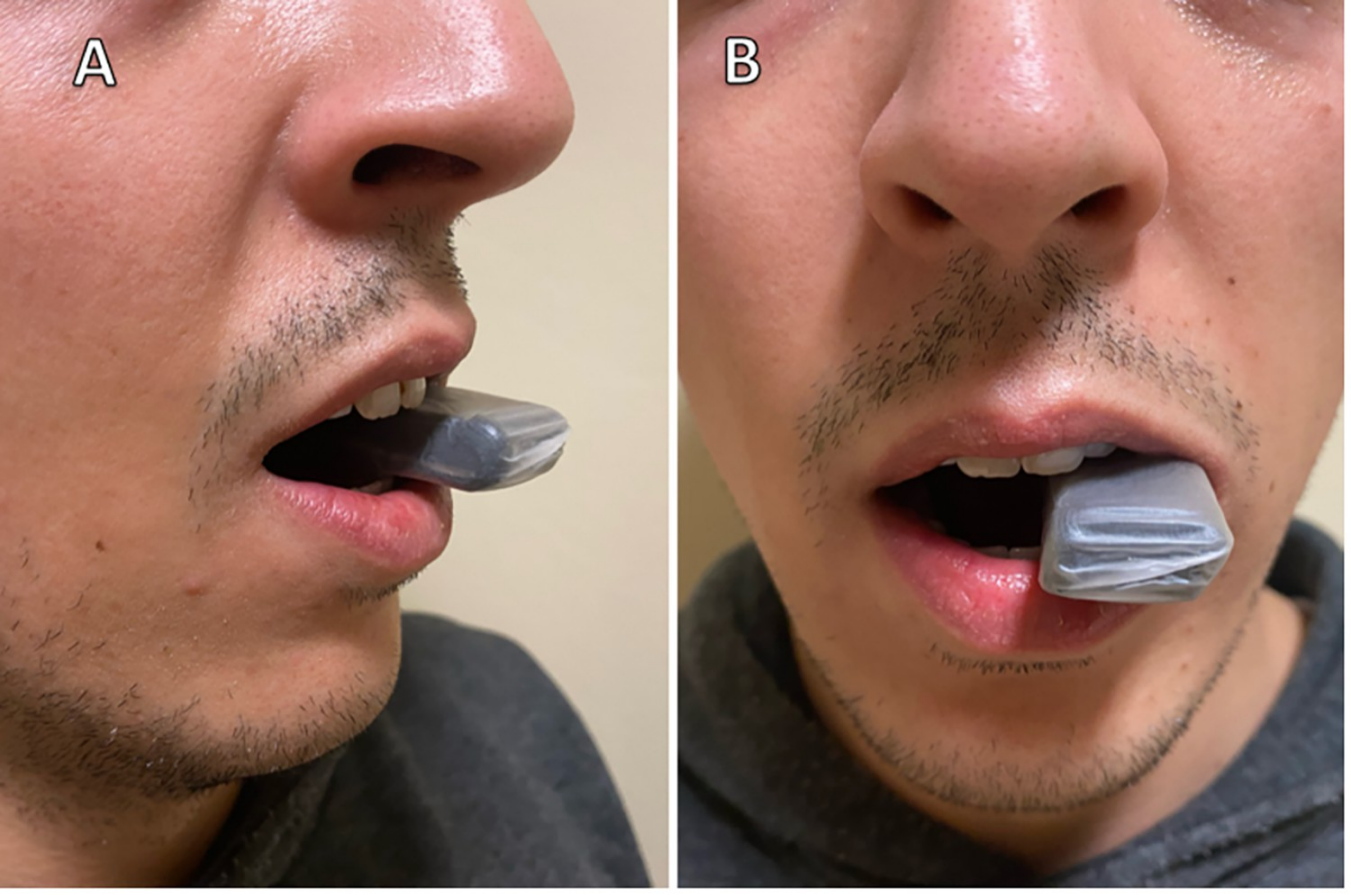
A 10-mm wedge is placed comfortably between the molar teeth during Mouth Open Swallowing Maneuver. A: Anterolateral view; B: Anterior view.

A 10-mm wedge was created by combining 6 wooden tongue depressors with tape. In the sitting position, the wedge was placed between the participant’s first molar teeth, and after the recording started, the participants were instructed to ‘hold the wedge with their teeth and swallow their saliva with their mouth open (Fig 2) (S1 Movie).

#### Mendelsohn maneuver (MM)

The MM is the method of voluntarily holding the larynx elevated for a while during swallowing [14]. After the recording started, the participants were instructed to ‘swallow and hold the larynx at the highest point for 3 seconds’.

#### Tongue-hold maneuver (THM)

The THM is to place the tongue between the front teeth and swallow saliva in this position [18]. After the recording started, the participants were instructed to ‘swallow their saliva with their tongue positioned between their front teeth’.

### Data collection

#### VFSS procedure

VFSS is a radiographic method in which spatial, temporal, and visual perceptual parameters of swallowing can be evaluated [31]. Hyoid and larynx move vertically and horizontally during swallowing to close the airway and open the UES. It also facilitates the tilt of the epiglottis with the contact of the larynx and floor of the mouth. The direction and amount of movement are determined by kinematic analysis with VFSS records. Hyolaryngeal kinematic analysis provides reliable quantitative assessments of swallowing [32]. In the current study, the VFSS (Siemens, Luminos Fusion FD, 31209, Germany) was recorded in the lateral plane (30 fps) while the participant was sitting upright on a chair in a comfortable position. Recordings included oral cavity, pharynx, larynx, cervical vertebrae, and upper esophagus. A coin (17 mm) was placed on each participants’ mastoid process using medical tape. The participants performed swallowing tasks as described above. All tasks were tested by only saliva swallowing without bolus.

**Fig 3:**
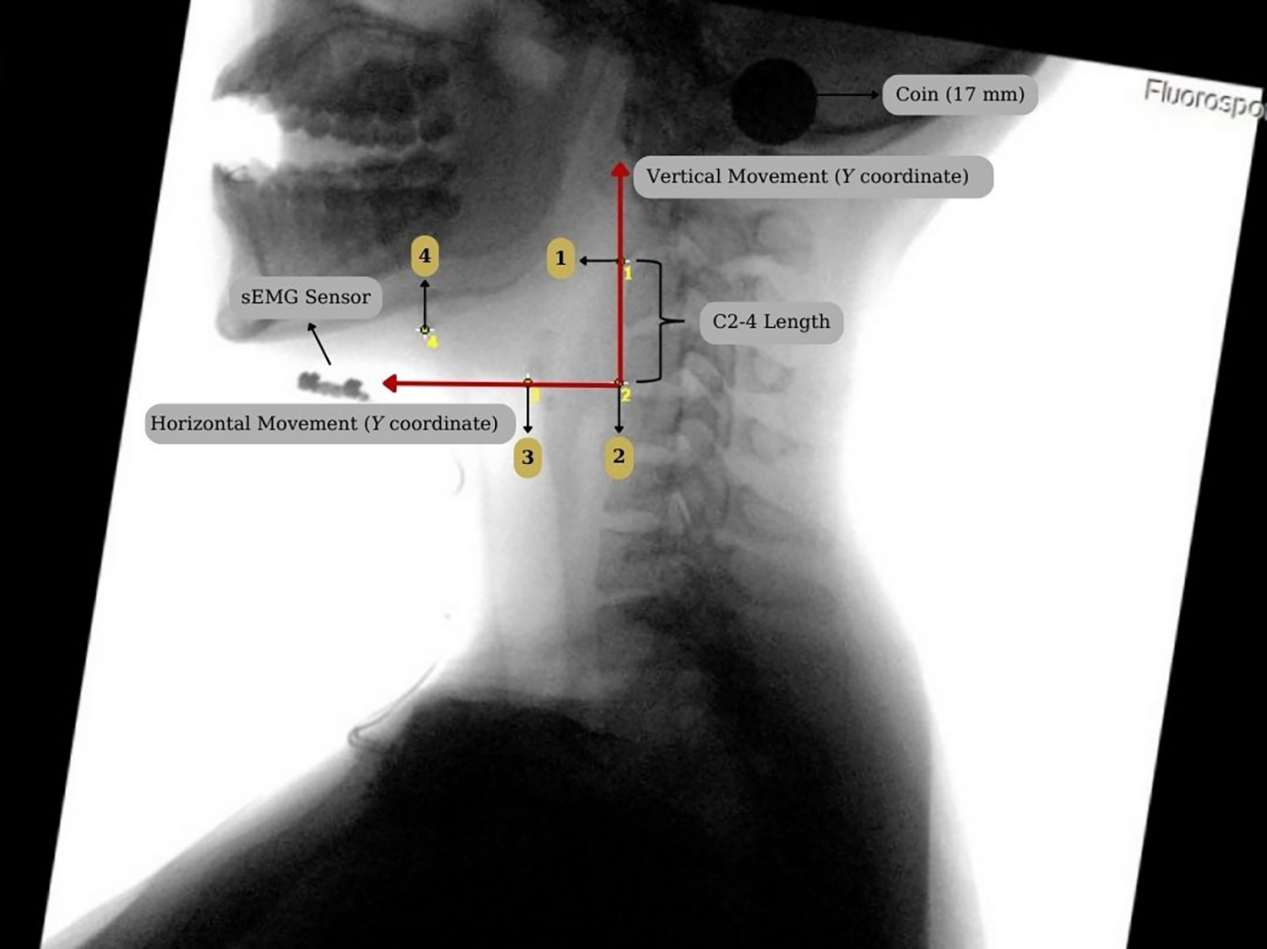
Kinematic analysis during Mouth Open Swallowing Maneuver (rotated picture). Anatomical Scalar: 1: anterior-inferior portion of C2; 2: anterior-inferior portion of C4; 3: posterior-superior portion of the subglottic air column; 4: anterior-inferior portion of hyoid.

VFSS recordings were analyzed frame-by-frame by a clinician who had experience in dysphagia management for 6 years. Recording of each participant was viewed. The first frame in which the hyoid and larynx started to move immediately after the participants were instructed was considered as the beginning of swallowing. The first frame was chosen as the last resting position of the hyoid and larynx just before the beginning of swallowing. Then, the frame in which the upward and forward movement of the hyoid and larynx during swallowing was in the highest position was chosen as the last frame [33, 34]. The first frame and the last frames for hyoid end larynx position were exported in an image format for kinematic analysis. All kinematic analyses were performed using ImageJ. First, the images were rotated so that C2 and C4 were in the same vertical alignment at 90 degrees. Thus, individual positional differences were standardized, and the horizontal and vertical movements of the hyoid and larynx were evaluated correctly in the X and Y planes on a pixel basis. Between the anterior-inferior point of C2 and C4 vertebrae, a straight line to measure C2-4 length. The diameter of the coin was measured in pixels. Anterior-inferior point of the hyoid, the posterior-superior point of the subglottic air column, and the anterior-inferior point of C2 and the anterior-inferior point of C4 were marked for each image. During the tasks, the anterior-inferior point of the hyoid and the posterior-superior point of the subglottic air column were used for the initial position of the hyoid and larynx, and their positions were determined relative to the anterior-inferior point of C4 [34–36] (Fig 3). The initial position of the hyoid and larynx for MOSM was measured with the mouth open. All pixel-based data were exported to MS Excel and analyzed. The diameter measurement of the coin was used to convert pixels to mm. To account for the sex factor, the amount of hyolaryngeal movement was given as the ratio of movement to C2-C4 length [37].

The intra-rater and inter-rater reliability of the kinematic analysis on 20% of the data were performed by the first and third authors.

#### Submental surface electromyography procedure

Submental surface electromyography (sEMG) was used to measure the amount of peak activation of swallowing muscles contraction during tasks. Submental sEMG reflects the SMA including the mylohyoid, geniohyoid, and digastric muscles [19, 38, 39]. sEMG and VFSS were recorded synchronously during all swallows. Before the measurement of sEMG, the skin was cleaned with an alcohol wipe. After drying the skin (30 sec), Delsys Inc. Trigno Duo sEMG wireless sensors were placed under the chin on the right and left sides of the midline for the submental muscles [40]. Sampling size was 1037 Hz. The raw signal was band-pass filtered (20–450 hz) and rectified with 0.05 s window length using the Root Mean Square method.

Before all assessments, participants wore a semi-rigid neck orthosis for the measurement of the maximum voluntary contraction (MVC) of the submental muscle with sEMG recording [39, 41]. The participant was asked to open their mouth as much as they could for 5 seconds against the anterior part of the orthosis. The measurement was performed 3 times. The maximum value was used for normalization. The sEMG activations of the exercises are given in proportion to the MVC [39].

### Statistical analysis

Effect size (d) was determined as 0.9 in the post-hoc analysis using G-Power version 3.1 using the DS and MOSM data of 50 participants. The power of the study was greater than 95% in the power analysis with this effect-size. Statistical analyses were performed using the SPSS software version 25 (SPSS Inc., Chicago, IL, USA). The variables were investigated using visual (histograms, probability plots) and analytical methods (Shapiro-Wilk test) to determine whether they distributed normally. The variables were not found as normal distribution. Friedman tests were conducted to test whether there is a significant difference between tasks in term of hyolaryngeal movement and SMA. The Wilcoxon test and Paired Samples t-test were performed to test the significance of pairwise differences using Bonferroni correction to adjust for multiple comparisons (Bonferroni correction for multiple comparisons: 0.05/6 = 0.0083). An overall 5% type-I error level was used to infer statistical significance.

The intra-rater and inter-rater reliability of the kinematic analysis on 20% of the data were performed with intra-class correlation coefficients (ICC). In the evaluation of the results, values below 0.5 were considered as poor reliability, values between 0.5 and 0.75 as moderate reliability, values between 0.75 and 0.9 as good reliability and values above 0.9 as excellent reliability [42].

## Results

Fifty healthy volunteers were included in the study, twenty-two of the participants were male (mean age 23.27±4.16 years) and twenty-eight were female (mean age 22.71±2.99 years). The mean C2-4 length was 29.61±2.90 mm.

Intrarater and interrater reliability of kinematic analyses are presented in Table 1.

**Table 1:** Intrarater and interrater reliability of kinematic analysis.

	Hyoid Movement		Laryngeal Movement	
	Vertical *y* coordinate	Horizontal *x* coordinate	Vertical *y* coordinate	Horizontal *x* coordinate
Intrarater reliability	ICC (95% CI)	ICC (95% CI)	ICC (95% CI)	ICC (95% CI)
**Dry Swallowing**	0.98 (0.92–0.99)	0.96 (0.83–0.99)	0.86 (0.45–0.96)	0.93 (0.73–0.98)
**Tongue-Hold Maneuver**	0.96 (0.84–0.99)	0.95 (0.68–0.98)	0.73 (0.04–0.93)	0.90 (0.62–0.97)
**Mendelsohn Maneuver**	0.95 (0.84–0.98)	0.94 (0.78–0.98)	0.73 (0.05–0.93)	0.91 (0.67–0.97)
**Mouth Open Swallowing Maneuver**	0.96 (0.88–0.99)	0.95 (0.82–0.98)	0.87 (0.47–0.96)	0.87 (0.48–0.96)
**Inter-rater reliability**				
**Dry Swallowing**	0.84 (0.36–0.96)	0.94 (0.75–0.98)	0.81 (-0.63–0.95)	0.54 (-0.46–0.87)
**Tongue-Hold Maneuver**	0.94 (0.77–0.98)	0.87 (0.51–0.96)	0.46 (-1.00–0.86)	0.68 (-0.11–0.92)
**Mendelsohn Maneuver**	0.93 (0.71–0.98)	0.84 (0.36–0.92)	0.69 (-0.44–0.93)	0.79 (0.11–0.95)
**Mouth Open Swallowing Maneuver**	0.97 (0.91–0.99)	0.94 (0.78–0.98)	0.88 (0.55–0.97)	0.67 (-0.15–0.91)

ICC: Intra-class correlation coefficient; CI: Confidential interval

### Initial position of the hyoid and larynx

The initial positions of the hyoid and larynx for the tasks are given in Table 2. The initial positions of the hyoid and larynx during the tasks were compared. There were significant differences between tasks in terms of the initial positions of the hyoid and larynx in the vertical plane (0.004 and 0.001). In pairwise comparisons, the vertical initial position of the hyoid for MOSM was lower than DS, THM, and MM (0.002, 0.002, 0.001, respectively). The vertical initial position of the larynx for MOSM was lower than DS, THM, and MM (0.006, 0.004, p<0.008, respectively). No difference was found between the tasks in terms of the initial position of the hyoid and larynx in the horizontal plane (p = 0.926, p = 0.241).

**Table 2:** Initial positions of the hyoid and larynx relative to C4.

		DS	THM	MM	MOSM	x^2^	p*
		Mean (mm) ± SD	Mean (mm) ± SD	Mean (mm) ± SD	Mean (mm) ± SD		
**Hyoid**	Horizontal *x* coordinate	31.85±3.66	31.80±3.66	31.94±3.63	32.01±3.89	0.466	0.926
	Vertical *y* coordinate	12.34±6.31	12.31±6.26	12.59±6.80	9.85±7.90	13.201	**0.004**
**Larynx**	Horizontal *x* coordinate	11.42±2.15	11.13±2.25	11.27±2.12	11.52±2.07	4.201	0.241
	Vertical *y* coordinate	-13.85±6.95	-13.44±6.66	-12.89±7.27	-15.22±7.54	16.851	**0.001**

DS: Dry Swallowing, TMH: Tongue-Hold Maneuver, MM: Mendelsohn Maneuver; MOSM: Mouth Open Swallowing Maneuver

p*: Friedman test, p<0.05; df: 3

### Maximum position of the hyoid and larynx during tasks

The maximum positions of the hyoid and larynx during the tasks are given in Table 3. There were significant differences between the tasks in terms of the maximum position of the hyoid and larynx in the vertical and horizontal planes (Table 3). In pairwise comparisons, the vertical maximum position of the hyoid for DS was lower than THM (p = 0.001). The horizontal maximum position of the hyoid for MM was lower than DS, THM, and MOSM (p<0.008, p<0.008, p<0.008). The vertical maximum position of the larynx for MOSM was higher than DS and MM (p<008, p = 0.003). The vertical maximum position of the larynx for THM was higher than DS (p<008). The horizontal maximum position of the larynx for THM was higher than MM (0.002).

**Table 3:** Maximum positions of the hyoid and larynx relative to C4 during tasks.

		DS	THM	MM	MOSM	x^2^	p*
		Mean (mm)±SD	Mean (mm)±SD	Mean (mm)±SD	Mean (mm)±SD		
**Hyoid**	Horizontal *x* coordinate	39.21±4.48	39.19±3.64	37.51±4.30	38.98±4.52	31.00	0.000
	Vertical *y* coordinate	22.47±6.42	23.80±6.71	24.83±9.07	23.91±6.90	11.51	0.009
**Larynx**	Horizontal *x* coordinate	14.45±2.26	14.54±2.56	13.80±2.28	14.18±2.19	8.29	0.04
	Vertical *y* coordinate	5.51±6.27	6.86±6.12	5.93±7.07	7.81±5.80	23.42	0.00

DS: Dry Swallowing, TMH: Tongue-Hold Maneuver, MM: Mendelsohn Maneuver, MOSM: Mouth Open Swallowing Maneuver

p*: Friedman test, p<0.05; df: 3

### Hyolaryngeal movement during tasks

The median and minimum-maximum values of movements of the hyoid and larynx in the vertical and horizontal planes during tasks are given in Table 4. There were significant differences among tasks in vertical and horizontal movement of the hyoid and in vertical movement of the larynx (p<0.001, p = 0.008, p<0.001, respectively). There was no significant difference in horizontal movement of the larynx (p = 0.785).

**Table 4:** Hyolaryngeal movement and SMA during tasks.

		DS	THM	MM	MOSM	x^2^	p*
		Median (min-max)	Median (min-max)	Median (min-max)	Median (min-max)		
**Hyoid Movement^a^**	Vertical *y* coordinate	35.18 (5–75.29)	37.86 (11.25–66.46)	37.52 (8.01–82.47)	46.97 (18.9–84.71)	20.28	**0.000**
	Horizontal *x* coordinate	24.33 (3.41–48.78)	22.99 (4.17–51.85)	19.56 (-1.86–43.56)	21.84 (1.49–47.56)	11.81	**0.008**
**Larynx Movement** ^**a**^	Vertical *y* coordinate	63.79 (41.11–101.18)	68.77 (45–101.17)	61.34 (40.7–108.24)	72.62 (50–137.65)	53.09	**0.000**
	Horizontal *x* coordinate	9.48 (-5.13–23.17)	10.24 (-3.95–46.91)	8.66 (-3.85–26.92)	8.74 (-3.85–20.73)	1.06	0.785
**SMA**		10.87 (1.43–38.76)	10.31 (3.39–44)	10.28 (1.4–51.9)	7.37 (1.80–34.73)	9.45	**0.024**

^a^Movement: C2–C4% length; SMA: Submental Muscles Activation (MVC%)

p*: Friedman test, p<0.05; df:3

The vertical movement of the hyoid during the MOSM was significantly greater than those observed during DS and the THM (p<0.001, p<0.001). No significant difference was found between other movements in the vertical plane (p>0.008). Horizontal movement of the hyoid during DS and the THM were significantly greater than that observed during the MM (p = 0.001, p = 0.001). No significant difference was found between other movements in horizontal plane (p>0.008).

In the vertical movement of the larynx, the MOSM was significantly greater than DS, the MM, and the THM (p<0.001, p<0.001, p<0.001). In addition, vertical movement of the larynx during the THM was significantly greater than those observed during DS and the MM (p = 0.004, p = 0.002, respectively). There was no significant difference between DS and the MM in vertical movement of the larynx (p = 0.322). There was no significant difference between tasks in horizontal movement of the larynx (p = 0.785).

### Submental sEMG activity during tasks

The median and minimum-maximum values of SMA during tasks are given in Table 4. There were significant differences among tasks (p = 0.024). In the post-hoc analysis, the difference was found only between THM and MOSM. SMA during the THM was significantly greater than that observed during the MOSM (p = 0.002). No significant differences were found in other pairwise comparisons between tasks (p>0.008).

## Discussion

In this current study, the MOSM was designed as a new maneuver to improve hyolaryngeal movement, and both biomechanical and electromyographic properties of the MOSM were investigated by comparing with the DS, THM and the MM. Accordingly, the MOSM revealed more vertical hyoid movement than DS and the THM. DS showed more horizontal hyoid movement than the MM and the THM. The MOSM revealed more vertical larynx movement than DS, THM and MM. The THM also showed more vertical larynx movement than DS and the MM. No difference was found among tasks in horizontal larynx movement. Only THM showed greater activation in the SMA than MOSM during these tasks, whereas there was no difference in the SMA between the other tasks.

The first hypothesis of the study was that more hyolaryngeal movement would occur during MOSM because the hyoid moves downward and backward during mandibular depression. The vertical hyoid and larynx movements were greater during MOSM compared to DS, THM, and MM. MOSM was not superior to the other tasks in the horizontal hyolaryngeal movement. These results partially confirm our first hypothesis. In the hyolaryngeal complex, the tongue, jaw, and hyoid are associated with muscles and tendons. For example, the hyoid muscles attach the hyoid bone to the jaw and skull, and the tongue is attached to the jaw and hyoid by the genioglossus and mylohyoid muscle. Due to the kinematic linkage of the tongue, jaw, and hyoid, their movement may affect each other [43, 44]. Muto et al. reported that the hyoid moves down and backward at maximum mouth opening [28]. In our study, it was shown that the initial position of the hyoid and larynx was lower only vertically with mandibular depression during MOSM compared to DS, THM, and MM. This provided a greater range of motion for vertical hyolaryngeal movement. The reason for the lack of difference in the horizontal initial position of the hyoid and larynx may be due to insufficient mandibular depression.

The second hypothesis of our study was that submental muscle activity would also increase due to more hyolaryngeal movement during MOSM. The MOSM revealed more hyolaryngeal movement and similar SMA compared to DS and the MM and less SMA compared to the THM. The results of the present study did not confirm our second hypothesis. However, due to the tongue, jaw, and hyoid linkage, submental muscle activity reflects not only the activity of the muscles that provide hyolaryngeal movement but also the activity of the muscles involved in tongue movements [45, 46]. The pressure created by tongue-palate contact during swallowing provides the propulsive force required for the oral phase. Reis et al. reported that tongue pressure is related to SMA. As tongue pressure increases during swallowing, more SMA is generated [47]. As it can be understood observationally during MOSM, palatal contact with the anterior part of the tongue does not occur. The greater vertical hyolaryngeal movement during MOSM may have caused an increase in SMA. However, the decrease in tongue pressure due to insufficient tongue-palate contact may have caused the total activity that should have increased during MOSM to remain unchanged. Besides, the long pharyngeal muscles may have contributed to vertical hyolaryngeal movement during MOSM. The primary muscle group that provides hyolaryngeal movement is considered to be the submental muscles. However, the role of posterior digastric, stylohyoid, thyrohyoid, and long pharyngeal muscles (stylopharyngeus, salpingopharyngeus, and palatopharyngeus), which are the other muscles associated with swallowing function, are still subjected to investigation [48, 49]. Pearson et al. examined the contribution of muscles related to the hyolaryngeal complex to swallowing function. According to the two-sling theory, while the submental muscles pull the hyolaryngeal structure anteriorly with the help of the thyrohyoid muscle, the long pharyngeal muscles (stylopharyngeus, salpingopharyngeus, and palatopharyngeus) pull it posteriorly [49]. Opening the mouth during the MOSM may have affected the force vector of the submental muscles, which complicates completing the movement. Therefore, the reason why SMA during the MOSM remained similar with DS may be because the long pharyngeal muscles compensated the submental muscles and contributed hyolaryngeal movement. The fact that the amount of movement has increased in vertical direction in the present study also strengthens this claim. In future studies, this interpretation can be made clearer by evaluating the activation of the thyrohyoid and long pharyngeal muscles during the MOSM.

Although the position of the hyoid and larynx decreased with mouth opening, much more movement was produced in the vertical plane during the MOSM compared to other tasks. The SMA was similar to DS and the MM, but less than the THM. During MOSM, more vertical hyolaryngeal movement elicited with similar or less muscle activation. Increased performance of the submental muscles can also be explained by muscle architecture. Muscle architecture describes the organizational parameters of the muscle such as fiber length and physiological cross-sectional area. In recent years, muscle architectural features have been frequently used to understand muscle-joint behavior, to make surgical decisions and to develop exercises [50, 51]. Skeletal muscle architecture is one of the most important properties that determines a muscle’s force and excursion capability. The sarcomere is a functional part of muscle contraction and it contains contractile filaments such as actin and myosin [52]. Muscle contraction is generated by the sliding and overlapping of actin and myosin filaments. The increase in sarcomere length results in a decrease in the contact between actin and myosin fibers, resulting in a reduced capacity of the muscles to generate force [52, 53]. Long sarcomere length becomes relatively shorter with positional correction. As a result, the actin and myosin interaction increases and can generate more muscle strength [54]. It was reported that the optimum sarcomere length, which is 2.3 μm, can extend up to 2.6 μm under passive tension [53]. The sarcomere of the swallowing muscles is generally long (Mylohyoid, Digastric, Stylohyoid) [55, 56]. During the MOSM, the suprahyoid muscles are placed in a short position, in which the sarcomere length may be close to the optimum value. This may have increased muscle performance which may contribute to muscle activation.

Wheeler-Hegland et al. evaluated the SMA and hyoid movement of control swallowing, the MM, effortful swallow, and Expiratory Muscle Strength Training with 10 ml thin-liquid barium. SMA was found to be higher than control swallowing during the MM. Maximum hyoid movement was not different between control swallowing, the MM, and effortful swallow. However, the maximum angle of hyoid movement was higher for the MM than control swallowing. These results were interpreted as the vertical movement of the hyoid may have increased [57]. Inamoto et al. reported significant differences in vertical movement of the hyoid between the MM and control swallowing using 4-ml nectar-thick liquid [15]. We found that there is no difference between DS and the MM in terms of SMA and hyolaryngeal movement. The most important reason for this is that, unlike our study, bolus swallowing was performed during the maneuvers in these studies. Previous studies have reported the effect of bolus on swallowing kinematics [58–60]. Certainly, the amount of bolus is important to change swallowing kinematics as well. Swallowing of a 20 ml of liquid bolus was reported to increase the maximum movement of the hyoid according to 5 ml and 10 ml [60]. The swallowing of the bolus during the THM has potential risk factors, such as an increased risk of aspiration in some patients, increased pharyngeal residue, a shorter duration of airway closure, and a delay in the initiation of the pharyngeal swallow [18]. Therefore, the THM was thought as a therapeutic maneuver rather than compensatory maneuver [21]. For that reason, we designed the MOSM as a therapeutic maneuver. Therefore, all tasks were performed without the bolus.

The THM has been defined as a maneuver that focuses on increasing contact of the tongue base with the posterior pharyngeal wall [18]. In this study, we showed that the THM also contributes to the hyolaryngeal movement. Compared to the MM, the THM showed more horizontal movement of the hyoid, and in vertical movement of the larynx compared to DS. While providing these movements, it showed similar SMA with DS and the MM. Fujiwara et al. showed that the load on the submental muscles varied in different tongue protrusions. SMA during the THM (2 cm tongue protrusion) was higher than the THM (1 cm tongue protrusion) and DS [19]. In the present study, we asked participants to comfortably position their tongue between the teeth [61]. It is possible that we could not achieve 2 cm tongue protrusion. According to these studies, tongue protrusion below 2 cm may not be difficult to change the amount of load on the submental muscles. In addition, the THM showed more hyolaryngeal movement than DS and the MM in the current study. As the protrusion of the tongue increases during THM, the load of the suprahyoid muscles and resistance to tongue retraction increases. Fixation of the anterior part of the tongue may also increase the activity of the tongue root to compensate for swallowing. Matsuo et al. found that jaw position was associated with the position of the anterior part of the tongue, while hyoid position was associated with the position of the posterior part of the tongue. According to the study, tongue activity may affect the hyoid position. During swallowing, extrinsic tongue muscles such as genioglossus and palatoglossus stabilize the dorsal surface of the tongue against the hard palate, while hyoglossus may pull the hyoid upward [62]. The increase in tongue root activity during THM may have contributed to the hyolaryngeal movement being more prominent than in DS and MM.

The strength of the present study is that this is the first research article of the newly developed MOSM evaluating the kinematic and electrophysiologic properties of DS, MOSM, THM, and MM, simultaneously. Thus, the contribution of the SMA to the hyolaryngeal movement was more clarified. Kinematic analysis consists of many potentially error-prone steps, including frame capture, determining reference points, converting each point to new x and y coordinates, and normalizing the data. Therefore, validation of the swallowing kinematic analysis is necessary for a precise interpretation of the results [63]. For that, the ICC measurements were performed in detail for intra-rater and inter-rater reliability of vertical and horizontal hyolaryngeal movement data during the tasks. Inter-rater reliability was moderate to excellent. Most of the inter-rater reliability was also moderate to excellent. However, inter-rater reliability was poor only for the vertical laryngeal movement of the THM. Although our results were largely compatible with the literature, the ICCs of some data were lower than those reported in other studies [35, 64, 65]. The potential margin of error in the interpretation of the hyolaryngeal kinematic analysis for vertical laryngeal movement of the THM should be taken into consideration.

A limitation of this study is the lack of electromyographic properties of the thyrohyoid and long pharyngeal muscles, which are thought to be important for hyolaryngeal movement. Diseases can change the physiology of swallowing [66, 67]. It can also be investigated how the MOSM affect problems such as decreased hyolaryngeal elevation, insufficiency in UES opening, and inadequate tongue-palate contact in patients with dysphagia. In the elderly population, swallowing physiology may change and adaptation to maneuvers may become difficult. Therefore, its effect in the elderly population should also be investigated [68]. In the current study, we examined immediate kinematic and electromyographic changes during the MOSM. The possible contribution of the long-term application to recovery in dysphagic patients should also be investigated.

## Conclusion

In conclusion, we designed the MOSM as a new maneuver that has the potential to be used in dysphagia rehabilitation to improve hyolaryngeal movement. This study showed that mouth opening increases the range of motion by lowering the hyoid and larynx and that MOSM is superior to other tasks by increasing the hyolaryngeal vertical motion more than this lowering. In addition, the THM is as effective as MM in hyolaryngeal movement. Furthermore, the fact that the MOSM had more hyolaryngeal movement with the same muscle activation provided important data on the contribution of the muscles of the hyolaryngeal complex to movement. In future, the clinical significance of the MOSM should be tested with studies involving patients.

## Supporting information

S1 Movie Practicing the Mouth Open Swallowing Maneuver. (MOV)

S1 Raw data (XLSX)
